# Advancements of radiotherapy for recurrent head and neck cancer in modern era

**DOI:** 10.1186/s13014-023-02342-0

**Published:** 2023-10-06

**Authors:** Shu Zhang, Ni Zeng, Jiangping Yang, Jinlan He, Fubin Zhu, Wenjun Liao, Maoqi Xiong, Yan Li

**Affiliations:** 1 Department of Head and Neck Oncology, Cancer Center, West China Hospital, SCU, Chengdu, Sichuan China; 2 Department of Radiation Oncology, Cancer Center, West China Hospital, SCU, Chengdu, Sichuan China; 3 Department of Oncology, Chengdu Seventh People’s Hospital (Affiliated Cancer Hospital of Chengdu Medical College), Chengdu, China; 4 Department of Radiation Oncology, Radiation Oncology Key Laboratory of Sichuan Province, Sichuan Clinical Research Center for Cancer, Sichuan Cancer Center, Sichuan Cancer Hospital& Institute, Affiliated Cancer Hospital of University of Electronic Science and Technology of China, Chengdu, Sichuan China; 5 West China Clinical Skills Training Center, West China School of Medicine, West China Hospital, SCU, Chengdu, Sichuan China; 6 Lung Cancer Center, West China Hospital, SCU, Chengdu, Sichuan China

**Keywords:** Head and neck cancer, Recurrent, Radiotherapy, Radiation techniques

## Abstract

Head and neck cancer is a kind of cancer which can be eradicated from radical radiation therapy. However, with best efforts, nearly 40% patients will experience locoregional recurrence. Locoregional recurrence is the main cause of cancer-related death in head and neck cancers, so local treatments play a key role in improving progression free survival. In the last decades, radiation techniques have been tremendously developed, highly conformal radiation techniques such as intensity-modulated radiotherapy, stereotactic body radiation therapy, brachytherapy and proton or heavy ion radiation therapy have their unique radiobiological advances. Although reirradiation is widely used in clinical practice, but little is known when comparing the different techniques. In this review, we will provide a comprehensive overview of the role of reirradiation in recurrent head and neck cancers including radiation techniques, patient selection, overall clinical benefits, and toxicities.

## Introduction

Head and neck squamous carcinoma (HNSCC) is a common head and neck malignancies, compromising of 4% in all the malignant tumors, and almost 67,000 newly diagnosis each year [1]. Nowadays, for the early stage HNSCC patients, surgery and definitive radiotherapy (RT) result in similar local control and survival. Postoperative RT with or without concurrent chemotherapy is indicated for those with pathological risk factors, i.e. perineural invasion, lymphovascular invasion,positive or close margin, et al. Locoregionally advanced HNSCC is associated with poorer survial, combined modality approaches (surgery, RT, and/or chemotherapy) are generally required to optimized the chances for long-term control, organ conservation and better quality of life [2–4]. So, RT is widely used in HNSCC treatment [5, 6]. However, approximately 40% patients will develop a recurrence within 5 years after definitive treatment. In addition, 15–40% survivors will be at risk of a secondary primary tumor in the irradiated area [7]. Moreover, recurrent cancer is the major factor contributing to cancer-related mortality.

In the past, surgery is first treatment for the recurrent head and neck cancers, sometimes completed resection is not feasible due to the tumor extension to critical organs or patient condition is not suitable for surgery [8]. So radiotherapy is an alternative in recurrent head and neck cancers as a definitive treatment which can improve the clinical outcomes and cure rates [9].

Reirradiation is becoming more acceptable as for the highly conformal techniques, such as intensity modulated radiotherapy (IMRT), volumetric modulated arc therapy (VMAT), stereotactic body radiotherapy (SBRT), image-guided radiotherapy (IGRT) and protons or heavy ions. Indications for reirradiation were 1). adjuvant RT with or without chemotherapy after surgery with certain risk factors, i.e. close or positive margin, residue disease, extracapsular extension (ECE), et al.; 2). definitive RT for those patients who were not surgery candidates and 3). palliative RT, such as relieving pain. Speical consideration should be paid to the interval between two radiations must be at least over 6 months, better to be longer than two year, and the dose constrains to OARs [10]. A major drawback of reirradiation is the acute or late toxicity with dosage limitation to the surrounding organs at risks (OARs). Previously studies showed serious late treatment complications up to 30% [11–13].

In this study we will illustrate the survival benefit and treatment-related toxicities from different radiation techniques. Thus, we aim to give a comprehensive review of the radiation techniques which are being used in clinical scenario.

## External beam radiotherapy

### Dose escalation

IMRT and VMAT are two kinds of intensity modulated conformal radiotherapy which are widely used in head and neck cancers and have been extensively studied in reirradiation. The clinical application of IMRT or VMAT improves the locally tumor control and reduce the toxicities with more efficient dose delivery to the highly conformal area.

Several studies have investigated the relationship between dose and survival clinical outcomes. Balermpas et al. reported the survival outcomes and toxicities in 18 inoperable rHNSCC patients who received re-RT with total 50.4 Gy dose (1.8 Gy/fraction). The 1-year overall survival (OS) rate was 47% and 2-year OS rate was 19%. Median OS for all patients was 8.38 months. And the progression free survival (PFS) at 1 year or 2 year was 44% and 33%, respectively. The median PFS was 7.33 months. The maximum mild to moderate toxicities was 44% [14].Likewise, the other study also showed that reduced radiation dose (≤ 50 Gy) tend to be with impaired OS [15]. A large-scale multicenter analysis including 256 rHNSCC patients from Germany showed that re-RT of locally recurrent or second primary HNSCC is efficient, especially the treatment dose was above 50 Gy in which significantly resulted in better OS [16]. However, a study in 35 patients underwent re-RT with median dose escalation up to 55.8 Gy; the 2-year locoregional control (LRC) and 2-year disease free survival (DFS) rates were 9% and 7%, respectively. The 2-year OS rates were 7.9%. Grade 3 acute toxicity was reported in 7 (20%) patients while grade 3–4 late radiation-induced complications were seen in 8 (23%) patients [17].

Some experts also evaluated the effect of gross tumor dose up to 60 Gy (2 Gy/fraction). A study showed survival outcomes as 2-year OS rate ranging from 10 to 22%, 2-year DFS: 3–14%, but relatively higher incidence of treatment-related toxicity, with grade 3–4 acute toxicities ranging from 30 to 46%. However, these studies didn’t specifically mention which radiation technique was used at when 3DRT was the commonly used method [12, 18]. As in intensity modulated conformal radiotherapy period, the re-RT total dose of 60 Gy showed a trend with better OS and lower treatment-related toxicities. Rühle et al. found that either simultaneously integrated or sequential boost to gross tumor or tumor bed with a median total dose of 59.4 Gy would significantly improve OS [15]. In addition, local control rate is probably related to dose other than radiation techniques. Kakria et al. showed that by utilizing different techniques (IMRT vs. 3DRT) with total dose of 60 Gy, 3-year DFS and OS were not significantly different in both groups [19].

A multi-institution analysis in US investigated the correlation between dose and the outcomes. In this study, 52.7% of the patients treated postoperatively with gross disease (39/74), and 36.1% of those treated definitively received dose of ≥ 66 Gy. In both definitive RT group and postoperative subset with gross residue, higher dose resulted in improved OS and LRF. But the survival improvement in RT group might be eroded by competing risk of death due to distant progression or a non-head and neck cancer cause. On the contrary, adjuvant postoperative re-IMRT patients appeared to have little difference in either LRF or OS using doses of 50 to 66 Gy [20]. Takiar et al. also examined the role of the re-IMRT dose in a single-institution study, demonstrating an improvement in 5-year LRC, PFS, and OS with a dose of 70 Gy, compared with ≤ 66 Gy, for patients not receiving surgery [12].

### Conventional radiotherapy vs. surgery

Salvage surgery plays a key role in recurrent HNSCC treatment. With radical remove of tumor, patients can achieve long-term disease control rate (DCR) up to 45% [21, 22]. Moreover, in some circumstances, complete resection of early recurrent tumors, DCR may increase to 80% [23].

Indications for post-operative reirradiation are incomplete resection or ECE of nodal metastasis [24–26]. Several studies have showed that postoperative irradiation without concurrent chemotherapy in high-risk patients, the 3- and 4-year OS was observed in 48% and 43% [27] [28]. The efficiency and safety were further confirmed by a prospective randomized phase III trial. One-hundred-thirty recurrent HNSCC patients who received salvage surgery without macroscopic residue were randomly assigned into two arms: in the first arm, patients underwent observation and in the second arm patients underwent 3D-RT (median dose 60 Gy/30 fractions) conccurent with 5-fluorouracil (5-FU) and hydroxyurea. Patients in the observation arm had significantly worse LRC (P < 0.0001) and DFS (P < 0.01). Differences in OS did not reach statistical significance (P = 0.50). Thus, in this study the LRC or DFS didn’t indicate OS benefit. Meanwhile, 2-year severe toxicity rate was 39% in the post-irradiation arm compared to 10% in the observation arm (P = 0.06) [24].

A retrospective study from Mayo Clinic compared the difference between definitive reirradiation (DRRT) or postoperative reirradiation (PRRT). Eighty-one HNSCC patients received DRRT or PRRT, median PRRT and DRRT doses were 60 and 69.6 Gy, respectively. 95% patients received IMRT-based reirradiation. Two-year OS was 53% and 48% in PRRT and DRRT, respectively, (p = 0.12); local recurrent free survival (LRFS) at 2 years did not differ significantly between PRRT and DRRT groups, either. Late serious toxicities were uncommon, including 2 osteoradionecrosis and 1 non-fatal carotid artery bleeding [29].

Another study including 227 patients in which long-term survival was observed. 91% were retreated with either DRRT or PRRT. The reirradiation dose was higher for DRRT (median 66 Gy) versus PRRT (median 60 Gy) 0.104 patients (50%) underwent salvage resection, and 126 (73%) received radiotherapy, in which 116 (67%) received concurrent chemotherapy. Median OS was non-significantly different as for those DRRT was 27.7 months and 22.8 months for PRRT. And there is no significant difference in LRC at 5 years between surgery or non-surgery group (p = 0.27) [12].

A group from Belgium reported that 84 patients with rHNSCC were treated with IMRT to a median dose up to 69 Gy. 19 patients received salvage surgery followed by re-RT; 17 patients received concurrent chemotherapy. In the whole cohort, the median OS was 13.4 months. Actuarial OS at 1, 2 and 5 years was 54%, 35% and 20%, respectively. Median disease-specific survival (DSS) and DFS were 17.6 and 8.0 months, respectively. The 1-, 2- and 5-year DFS rates were 40%, 35% and 15%, respectively. DSS rate was 58%, 42% and 29% at 1, 2 and 5 years, respectively. OS and DFS was significantly worse in patients without salvage surgery [30].

### Conventional radiotherapy with systemic chemotherapy

Systemic chemotherapy has been used as a palliative method for patients who are not candidates for surgery, but the response rates are limited with a median survival time of 7.4 months [31]. Mounting of studies have evaluated the role of chemotherapy in treating HNSCC.

In 1998, a group from France [18] compared radiation alone with 5–6 cycles of fluorouracil (5-FU) and hydroxyurea concurrent chemotherapy. The median radiation dose prescribed was 60 Gy/30 f. Better objective response rate (ORR, 58% vs. 45%), 5-year OS rate (14% vs. 6%), 2-year DFS (14% vs. 11%), 5-year DFS (8% vs. 6%) were observed in chemoradiation group when compared with radiation alone, however, no statistical significance was shown. The incidence of grade 3/4 mucositis was 23% and 8% (radiation alone), 32% and 13% (chemoradiation). The two factors significantly correlated with survival were irradiated volume less than 650 cc or radiation dose more than 60 Gy.

Since then, different chemotherapy regimens were evaluated in rHNSCC patients. Hehr et al. [32] reported the clinical outcomes of concurrent chemoradiation in 27 inoperable rHNSCC patients. Alternating chemoradiation consisted of 3 cycles of docetaxel and cisplatin with involved field radiotherapy (Total dose 40.0 Gy/2.0 Gy per fraction). Grade 3 common toxicity criteria mucositis occurred in 15%, leukopenia ≥ grade 3 in 37%, and 3 early deaths were observed. Time to local progression, and 3-year OS rates were 10 months, and 18%, respectively. Other studies also showed that grade 3 or 4 acute toxicities occurred ranged from 20 to 46% mainly with mucositis, dermatitis and esophagitis of patients who received induction or concurrent chemotherapy with cisplatin or carboplatin [33, 34]. Concurrent chemoradiation with capecitabine was also investigated in rHNSCC. The total irradiated dose was 50 Gy up to 60 Gy. The ORR was 68%. The median OS was 8.4 months. Grade 3 or 4 mucositis occurred in 4 patients and 1 patient, respectively. No grade 4 hematological toxicities were observed; 1 patient had grade 3 anemia [35].

Salama et al. reported that 115 previously irradiated HNSCC patients, 49, who had undergone surgical resection, followed by concurrent chemoradiation and 66, who were treated with definitive chemoradiation. The chemotherapy regimens were used: 5-fluorouracil and hydroxyurea concurrent with reirradiation (FHX) (n = 14), cisplatin plus FHX (n = 23), paclitaxel plus FHX (n = 42), gemcitabine plus paclitaxel and 5-fluorouracil concurrent with reirradiation (n = 26), and irinotecan plus FHX (n = 10). The whole cohort median OS and PFS was 11 and 7 months (range, 0.2-158.7), respectively. The 3-year OS, PFS, LRC, and distant metastasis free survival (DMFS) rate was 22%, 33%, 51%, and 61%, respectively. Though, multivariate analysis identified reirradiation dose, triple agent (cisplatin-, paclitaxel-, or gemcitabine-containing chemotherapy), and surgery before radiation as independently prognostic for OS, PFS, and LRC. However, 19 patients died of treatment-related toxicity, five of these of carotid hemorrhage. Caveats in this study is that the role of chemotherapy was not illustrated separately in non-surgical resection group [36].

A retrospective study previously mentioned that concomitant chemotherapy did not lead to improved OS or PFS rates (no chemotherapy vs. chemotherapy), and there was no survival benefit between patients receiving cisplatin or not [15]. Lee et al [37] also reported that in a whole cohort of rHNSCC patients treated with either 3DRT or IMRT combined with chemotherapy as definitive or adjuvant intention, the use of different regimens of chemotherapy didn’t significantly improve the OS or locoregional recurrent free survival (LRRFS).

In a subset of evaluating the role of adjuvant chemotherapy post-surgery, several studies showed that additional adjuvant chemotherapy didn’t bring survival outcome [29, 34].

For advanced recurrent tumor, the tumor infiltrated surrounding critical organs, definitive radiotherapy caused more severe acute or late treatment-related toxicity. Several studies aimed to explore the efficacy of chemotherapy as reduce the tumor burden. ORR ranged from 45 to 83.3% in different set of chemotherapy agents [38–41]. A phase II clinical trial explored two-cycle induction chemotherapy containing regimen of gemcitabine and pemetrexed brought 35% ORR rate before concurrent chemoradiation. However, the proneness to toxicity and death of induction chemotherapy didn’t result in better OS compared to concurrent chemoradiation [42]. Considering the treatment-related toxicity might compromise the survival outcomes, Yang et al. retrospectively analyzed if a two-regimen induction chemotherapy (PF vs. TPF) would improve the OS. It showed that the overall response rate (RR) was better in the TPF group (p = 0.005) than the PF group. And patients who received TPF, followed by surgery and concurrent chemoradiotherapy had better OS (p = 0.012) and PFS (p = 0.038) than concurrent chemotherapy with PF group [43].

### Conventional radiotherapy with target therapy

Cetuximab is an anti-EGFR antibody which has been used in concurrent radiotherapy in HNSCC and in combination of chemotherapy in metastatic HNSCC. Several studies have shown that by using cetuximab as concurrent regimen, the median OS was 8.3 to 18 months, and PFS ranged from 7.3 to 15 months. And the 1-year OS rate was about 40%, local control rate was from 33 to 61%. ORR ranged from 47 to 59% [14, 44, 45].

Dornoff et al. compared concurrent cetuximab with concurrent cisplatin. The study showed that 66 patients with rHNSCC were 1:1 assigned to received cetuximab or cisplatin-based chemotherapy concomitant with re-RT. The 1-year OS rates for cetuximab and chemotherapy were 44.4 and 45.5% (p = 0.352), respectively. At 1-year local control rate (LCR) were 46.4 and 54.2% (p = 0.625), free from metastases (FFM) rates were 73.6% and 81% (p = 0.842), respectively. Over grade 3 haematological toxicity occurred more often in the cisplatin group (p < 0.001), pain ≥ grade 3 was increased in the cetuximab group (p = 0.034) [46].

Thus, cetuximab as a substitution for those patients’ ineligible of cisplatin without compromising the clinical outcomes or increasing treatment-related toxicities.

### Conventional radiotherapy with immunotherapy

Immunotherapy, mainly, immune checkpoint inhibitors (ICIs) like PD-1 or PD-L1 blockade inhibitors, has emerged as a promising treatment option for many types of cancers. And its role in recurrent HNSCC has been evaluated recently. The role of immunotherapy in rHNSCC is disputable.

Immunotherapy was used in inoperable recurrent or metastatic HNSCC patients. And ICIs were introduced in second-line treatment by using different ICIs [47–49]. And the improvement in OS was 20-31% reduction in risk of death, and even higher ORR when compared with standard care of chemotherapy. KEYNOTE-048 showed that even pembrolizumab monotherapy was found to be non-inferior to EXTREME study, in terms of OS (11.6 mo vs. 10.7mo), and ORR was better in pembrolizumab plus FP and EXTREME groups other than pembrolizumab alone [50].

A retrospective of 10 rHNSCC patients were re-evaluated 60 Gy in 30 fractions with concurrent and maintenance nivolumab administration. One-year OS rate was 50% and the median OS was 11 months. One-year LPFS rates were 30%. Median LPFS was 8 months. OS and LPFS rates were not inferior to those of patients treated with concurrent cisplatin. No unexpected radiation-related toxicity occurred [51]. Based on previously studies, there are several ongoing clinical trials to further investigate the safety and efficacy in rHNSCC when combined with re-irradiation.

**Table 1:** Re-irradiation studies using EBRT

Study	Study design	No. of patients	Previous radiotherapy, Gy	No. of fractions, median	Total re-irradation dose, Gy	surgery	Systemic therapy	Follow up, mo	Efficacy	Severe toxicity
Crevoisier, 1998	R	169	65–70		median:60	NA	84.6% chemotherapy	NA	2-y OS: 10–25%;5-y OS:0–14%	Grade 3–4 acute toxicity: 46%
									2-y DFS: 3–14%;5-y DFS:0–8%	
Crevoisier, 2001	R	25	≥ 45		median: 60	100%	100% chemotherapy	66	4-y OS: 43%	Grade 3 acute toxicity: 40%
									2-y DFS:36%,5-y DFS:26%	
Hehr, 2005	P	27	60-77.4		40–50	/	100% chemotherapy	42	3-y OS: 18%; median OS:10 mo	Grade 3–4 acute toxicity: 40%
Langendijk,2006	P	34	NA		median:60	/	/	32	2-y LRC:27%	Grade 3–4 acute toxicity: 30%
									2-y OS:22%; median OS:13.2 mo	Grade 3–4 late toxicity: 23%
Lee, 2006	R	105	median:62		median:59.4	34.30%	71.5% chemotherapy	35	2-y LRRFS: 42%	Grade 3/4 acute toxicity: 23%/15%
									2-y OS:37%;median OS: 15 mo	Grade 3–4 late toxicity: 18.5%
Salama, 2006	R	115	median:67.5		median:64.8	42.60%	100% chemotherapy	67.4	3-y OS: 22%; median OS: 11 mo	Grade 4–5 toxicity: 18.3%
									3-y PFS: 33%; median PFS:7 mo	
Kasperts, 2006	P	39	NA		60–66	100%	/	32	3-y LRC: 74%	Grade 3–4 late toxicity: 36%
									3-y OS: 48%	
Janot, 2008	P	130	≥ 45		median:60	100%	RT group 96.7% chemotherapy		RT group: 3-y OS: 25%, 3-y LRC: 58%	RT group: grade 3–4 acute toxicity: 28%,grade 3–4 late toxicity: 26%
Duprez, 2009	R	84	median:69		median:69	22.60%	20.2% chemotherapy	19.8	2-y LRC: 48%, 5-y LRC: 40%	Grade 3 acute toxicity: 31.0%
									2-y OS: 35%, 5-y OS: 20%; median OS: 13.4 mo	Grade 3 late toxicity: 13.1%
Sher, 2010	R	35	median:67.5		median:60	17%	100% chemotherapy	27.6	2-y LRC:67%	Grade 3–4 toxicity: 32.81%(TPF); 13.79%(PF)
									2-y OS: 48%	≥ Grade 3 late toxicity:46%
Jensen, 2010	R	73	median: 66		median: 45	/	100% cetuximab	NA	median LRFS: 12.8 mo; median PFS: 8.6 mo; median OS: 12.5 mo (Re-RT)	Grade 3 late toxicity: 27.3%
Zwicker, 2011	R	10	median:66		median:50.4	40%	100% cetuximab	6.5 mo after reirradiation	1-y OS: 40%; median OS: 7 mo	Grade 3 acute toxicity: 10%
									1-y LC:61%, 1-y LRC:44%	Grade 3 late toxicity: 20%
Villaflor,2011	P	35	NA		60–70	61.00%	100% chemotherapy	45.6	1-y OS:43%;median OS: 9.8 mo	/
									1-y PFS: 20%;median PFS: 9.5 mo	
Peponi,2012	R	35	median:66		median:55.8	NA	NA	12.9	1-y LC: 41%, 2-y LC:9%	Grade 3 acute toxicity: 20%
									1-y DFS: 30%, 2-y DFS: 7%	Grade 3–4 late toxicity:23%
									1-y OS: 42.9%, 2-y OS: 7.9%	
Vormittag, 2012	P	31	NA		50–60	/	100% chemotherapy	NA	2-y OS:10%;median OS: 8.4 mo	Grade 3–4 toxicity: 12.9%
Balermpas,2012	R	18	55.8–72		median:50.4	/	100% cetuximab		1-y OS: 44%, 2-y OS: 19%; median OS: 8.38 mo	Grade2-3 toxicity:44%
Yang,2014	R	93	45–72		NA	71%	100% chemotherapy	14.2	OS: not reach (PF);OS: 32.2 mo(TPF). Grade3-4 toxicity:32.8%(TPF) PFS: 27.5 mo(PF);PFS: 29.5 mo(TPF) 13.79%(PF)	
Kakria,2015	R	31	median:70		median:60	13%	45.2% cetuximab or chemotherapy	20.6	3-y DFS: 28.7%;median PFS: 12.7 mo	Grade 3 acute toxicity: 32.3%
									3-y OS: 48.7%;median OS:24.8mo	
Dornoff, 2015	R	66	median: 64		median:50.4	24%	50% cetuximab	18.3	1-y OS: 44.4% (cetuximab), 45.5%(cisplatin)	Grade 3 toxicity: 57.6% (cetuximab), 51.5%(cisplatin)
							50% chemotherapy			
Curtis, 2016	R	81	median:66		median:60(post-surgical RT)	51.90%	74.1% chemotherapy or cetuximab	78.1	2-y OS: 48%; median OS:22 mo(RT)	NA
					median:69.6(definitive RT)				2-y LRFS: 60%; median LRFS: 54.7 mo	
Takiar,2016	R	227	median:65		median:60(post-surgical)	50%	67% chemotherapy	24.7 after re-irradiation	Median OS: 27.7 mo (definitive RT); median OS: 22.8 (post-surgical RT)	Grade 3 toxicity: 30%
					median:66(definitive)					
Caudell, 2017	R	505	NA		median:60	49.20%	77.40%	21.5	2-y OS(defiitive group): 49.3%(≥ 66 Gy),34.2%(60-65.9 Gy), 30.4%(< 60 Gy)	≥ 3 acute toxicity was 22.1%
									2-y LRF(defiitive group): 50.9%(≥ 66 Gy),67.5%(60-65.9 Gy)	≥ 3 late toxicity was 16.7%
Rühle,2020	R	48	median:68.2	30	median:59.4	35.40%	60.7% cetuximab	/	1-y OS: 62.4%, 2-y OS: 52.3%, 5-y OS: 34.3%; median OS: 25 mo	Grade 3 acute toxicity: 9.3%
							39.4% chemotherapy		1-y PFS: 37.6%, 2-y PFS: 28.8%, 5-y PFS: 11.5%; median PFS: 9 mo	Grade 3–4 late toxicity:16.6%
Altay-Langguth, 2021	P	10	median: 70.6(definitive) 64.8(adjuvant)		median:60	NA	100% Nivolumab	11	1-y OS: 50%; median OS:11 mo 1-y PFS: 30%; median PFS: 8mo	Grade 3 toxicity: 20% (Nivolumab)
Roesch, 2022	R	253	NA		median:50	/	71% chemotherapy	24.8	2-y OS: 29%; median OS: 13.2mo NA	
									2-y PFS: 19%; median PFS: 7.9 mo	

Abbreviations: OS, Overall Survival; LC, local control; LRC, locoregional control; DFS, disease free survival; LRFS, local recurrent free survival; LRRFS, locoregional recurrent free survival; PFS, progression free survival

## SBRT

Stereotactic body radiation therapy, or SBRT, is a method that delivers high irradiation dose precisely to tumor targets with a hypofractionated schema, typically ≤ 5 fractions which reduce the overall treatment time compared to conventional EBRT. Meanwhile, a steep dose gradient is created around target volume minimizing damage to healthy tissue [52]. SBRT are common in treating intracranial metastasis, spinal metastasis, and NSCLC [53–55]. The studies by using SBRT in recurrent HNSCC or secondary primary tumor were reviewed below.

### Dose modification in SBRT

In 2009, Heron et al. conducted a phase I dose-escalation trial in SBRT for rHNSCC. In this clinical trial, 25 rHNSCC patients were treated in five dose tiers up from 25 to 44 Gy. No Grade 3/4 or dose-limiting toxicities occurred. The ORR was 17%. Median PFS was 4 months, and median OS was 6 months. Self-reported quality of life was not significantly affected by treatment [56].

Roh et al. reported in 36 rHNSCC patients who received median dose of 30 Gy in 3–5 fractions by SBRT, achieved 88.6% ORR which resulted in a 2-year OS of 30.9%. Late adverse reactions occurred in 8.6% of patients including 2.9% treatment-related deaths [57]. Another analysis from 45 rHNSCC patients treated with median total dose of 30 Gy SBRT delivered in 5 fractions. The prescription doses of SBRT ≥ 40 Gy were associated with higher 1-year rates of OS (67.79%), LC (75.00%) and a higher likelihood of experiencing toxicities. Acute and late toxicity rates were low (22.2% and 15.6%, respectively) and were all Grade 1–2 with only one late Grade 3 esophagitis [58].

Kawaguchi et al. reviewed 22 patients who received SBRT for rHNSCC with restage rT1-rT4. The median marginal dose of 33.73 Gy in 2 to 5 fractions. When the PTV was more than 30 cc the prescription dose was reduced by 30%. Over 70% patients were observed ORR including CR and PR. The OS at 2-year with and without lymph node metastases is 12.5% and 78.6%, respectively. With higher recurrent T stage, there was a trend for more severe complications [59].

A group from University of Pittsburgh, reviewed 85 patients who received SBRT for rHNSCC. The mean dose of SBRT was 35 Gy. Those patients who received SBRT < 35 Gy had significantly lower LCR than those with ≥ 35 Gy at 6 months (P = 0.014). Tumor responses were 34% CR, 34% PR, 20% SD, and 12% PD. The 1-year and 2-year LCR and OS rates for all patients were 51.2% and 30.7%; 48.5% and 16.1%, respectively. Overall, the median OS for all patients was 11.5 months. Treatment was well-tolerated with no grade 4 or 5 treatment-related toxicities [60].

In 2018, a multi-institutional study compared the efficacy and safety between SBRT and IMRT for rHNSCC patients. The study included 414 patients with unresectable rHNSCC: 217 with IMRT and 197 with SBRT. The unadjusted 2-year OS rate was 35.4% for IMRT and 16.3% for SBRT (P < 0.01). The median OS were 13.3 months for IMRT patients and 7.8 months for SBRT patients. No significant differences in OS or locoregional failure between IMRT and SBRT were demonstrated. Acute grade ≥ 4 toxicity was greater in the IMRT group than in the SBRT group (5.1% vs. 0.5%, P < 0.01), with no significant difference in late toxicity. Further subset analysis demonstrated comparable OS when ≥ 35 Gy was delivered with SBRT to small tumor volumes (< 25 cc) [61].

To assess the effect of SBRT dose and tumor volume on the outcomes in patients with rHNSCC, Rwigema et al. reviewed 96 patients who received SBRT. Patients were divided into 4 SBRT dose groups: I (15–28 Gy/n = 29), II (30–36 Gy/n = 22), III (40 Gy/n = 18), and IV (44–50 Gy/n = 27). The median gross tumor volume (GTV) was 24.3 cm^3^. For GTV ≤ 25 cm^3^ (n = 50), complete response rates were 27.8%/30%/45.5%/45.5%, and for GTV > 25 cm^3^ (n = 46), complete response rates were 20%/25%/42.8%/50% in SBRT groups I-IV, respectively. The 1-/2-/3-year LRC rates for doses 40 to 50 Gy were 69.4%/57.8%/41.1%, respectively, whereas for 15 to 36 Gy, they were 51.9%/31.7%/15.9%, respectively (P = 0.02). The overall 1- and 2-year OS rates were 58.9% and 28.4%, respectively. The better LRC was observed in higher dose group related to smaller tumor volume [62]. Another retrospective study showed that for a curative intent for a rHNSCC patients who treated by SBRT with the median tumor dose was 30 Gy which were correlated to the median gross tumor volume of 58.7 cm^3^. However, there was a trend of lower response rate (25% CR, 31% PR and 44% SD) in relatively larger tumor volume [63].

### Adjuvant SBRT after salvage surgery

A retrospective review of 28 patients were with high-risk features (positive surgical margins or extranodal extension) following macroscopic complete (R0/R1) salvage surgery treated with adjuvant SBRT ± cetuximab. the 1-year LRC, distant control, DFS, and OS were 51%, 90%, 49%, and 64%, respectively. Rates of acute and late severe (≥ grade 3) toxicity were low at 0% and 8%, respectively [64].

Another retrospective study showed that 11 patients were treated with salvage surgery followed by SBRT. The median total dose was 40 Gy. The 2-and 4-year DFS were 62.3% and 41.6%, while the 2-and 4-year OS probabilities were 80.0% and 53.3%, respectively. Two (18.1%) patients had local failure in the SBRT field. Three (27.3%) patients had distant metastasis. The interval between previous radiotherapy and SBRT ≤ 24 months (p = 0.033) and location of the salvage target in the oral cavity (p = 0.013) were related to worse OS. The total dose of SBRT given in more than three fractions was favoring the OS (p = 0.051) [65].

### SBRT with systemic chemotherapy

A group from Georgetown University Hospital, reported a retrospective study in 65 rHNSCC patients by using SRS/SBRT. Thirty-eight patients were treated definitively and 27 patients with metastatic disease and/or untreated local disease were treated palliatively. Nine patients underwent complete macroscopic resection before SRS. Thirty-three patients received concurrent chemoradiation mostly choosing Xeloda, cisplatin, carboplatin as concurrent regimen. The median reirradiation SRS dose was 30 Gy (21–35 Gy) in 2–5 fractions. In all the 56 patients were evaluable for response: 54% CR, 27% PR, and 20% had no response. Median OS for all patients was 12 months. For definitively treated patients, the 2-year OS and LRC rates were 41% and 30%, Multivariate analysis indicated that higher total dose, surgical resection, and nasopharynx site were significantly associated with improved LRC; surgical resection and non-squamous histology were associated with improved OS. Seven patients (11%) experienced severe reirradiation-related toxicity, including one treatment-attributed death. However, the contribution of chemotherapy hadn’t been described in detail [66].

In 2015, Kress et al. reported a study evaluating the role of chemotherapy and long-term clinical outcomes in 85 rHNSCC patients by using SBRT. 60% patients received systemic chemotherapy including concurrent, adjuvant or induction chemotherapy. Results indicated that those patients treated with curative intent, the median OS was 12.2 months, and the 2-year OS and LRC were 24% and 28%, respectively. Median survival was 6.7 months in patients treated with palliative intent. Concurrent chemotherapy had no statistically significant impact on survival or LRC [67].

### SBRT with systemic target therapy

In a retrospective-matched cohort study, 70 rHNSCC patients were divided into 2 groups: patients with SBRT alone (n = 35) or with weekly cetuximab infusion during SBRT (n = 35). The concurrent cetuximab infusion conferred an OS advantage (24.5 vs. 14.8 months) when compared with the SBRT alone, without a significant increase in grade 3/4 toxicities [68].

A prospective study included 150 patients with unresectable rHNSCC were treated with SBRT of 40–50 Gy in 5 fractions, while 47% patients receiving concurrent cetuximab. The lifetime QOL including, swallowing, speech, saliva, activity was improved by SBRT regardless of age, tumor size or concurrent cetuximab [69].

Later, Lartigau et al. reported a muti-institutional phase II study of concomitant stereotactic reirradiation with cetuximab for rHNSCC, 60 patients with inoperable recurrent, or new primary tumor in a previously irradiated area were included. RT dose was 36 Gy in six fractions with 5 injections of concomitant weekly cetuximab. At 3 months, response rate was 58.4% and disease control rate was 91.7%. The one-year OS rate was 47.5%. Eighteen patients presented with grade 3 toxicities: mucositis, dysphagia, induration, and fibrosis and one toxic death from hemorrhage and denutrition [70]. Another phase II clinical trial held in US, concurrent cetuximab combined with radiotherapy, similar results were observed including the 1-year local PFS rate was 60%, and PFS was 33% (95% CI: 20-49%). The median OS was 10 months (95% CI: 7–16), with a 1-year OS rate of 40% (95% CI: 26-54%). Grade 3 acute and late toxicity was observed in 6% patients [71].

A group from Taiwan evaluated the role of SBRT in combination of cetuximab for Asian rHNSCC patients. The prescription dose ranged from 40 to 50 Gy in 5 fractions with concurrent weekly cetuximab infusion. The response rate was: 25.0%CR, 41.7% PR and 11.7%SD (n = 72) and 21.7%CMR, 51.7%PMR and 13.3% SMD based on PET-CT (n = 60), respectively. The median OS was 9 months and the 1-/2-year OS and PFS rates were 42.8%/22.0% and 40.5%/19.0%, respectively. The re-irradiation interval > 12 months and gross tumor volume (GTV) ≦ 50 ml were favorable prognostic factors of OS and PFS [72].

### SBRT with systemic immunotherapy

SBRT, different biological mechanism from conventional radiotherapy, is leading to cytotoxic effect and promote the tumor associated antigen release, thus, increasing T cell recognition and infiltration [73].

To date, there was no data presented in recurrent HNSCC. However, the only phase II randomized trial in metastatic HNSCC utilizing concurrent SBRT and nivolumab in which 62 patients with metastatic HNSCC was enrolled who were randomized to either nivolumab or nivolumab with SBRT (3 × 9 Gy). There was no statistically significant ORR difference between arms. There was no significant difference in OS or PFS. Grade 3–5 toxicities were similar [74]. Though the previous clinical failed to show any advantage in combination of SBRT with ICIs. However, it’s still worth to pursue other clinical trials ongoing to evaluate the efficacy of concurrent ICI with SBRT and identify the biomarker to predict prognosis in rHNSCC.

**Table 2:** Re-irradiation studies using EBRT

Study	Study design	No. of patients	Previous radiotherapy, Gy	No. of fractions, median	Total re-irradation dose, Gy	surgery	Systemic therapy	Follow up, mo	Efficacy	Severe toxicity
Heron,2008	P	25	median:64.7	5	25–44	/	NA	NA	17.4% CR + PR,48% SD	/
Roh, 2009	R	36	median:70.2	3 to 5	median: 30	/	11.1% chemotherapy	17.3	42.9% CR, 37.1% PR, 8.6%SD	Grade 3 acute toxicity:36.1%
									1-y OS:53.2%, 2-y OS: 30.9%	
									1-y LRS:61%, 2-y LRS: 52.2%	
Rwigema,2010	R	85	median:74	1 to 5	median:35	NA	NA	6	34% CR, 34%PR, 20%SD No grade 4–5 toxicities	
									1-y OS: 48.5%; 2-y OS:16.1%, median OS: 11.5 mo	
									1-y LC:51.2%; 2-y LC:30.7%	
Unger, 2010	R	65	median:67	5	median:30	13.80%	50.8% chemotherapy	16	54% CR, 27% PR Grade 4 toxicity:9%	
									2-y OS:41% median OS: 12 mo	
									2-y LRC: 30%	
Kawaguchi, 2010	R	22	40–65	2 to 5	20–42	/	100% chemotherapy	24	64.3% CR, 7.1% PR, 7.1% SD Grade 3 toxicity: 22.7%	
Heron, 2011	R	70	68–70	5	median:40	/	50% cetuximab	21.3(SBRT)	SBRT only: 2-y OS: 21.1%; median OS: 14.8 mo	Grade 3 acute toxicity: 14.3% (SBRT only)
								24.8(SBRT + cetuximab)	SBRT + cetuximab: 2-y OS: 53.3%; median OS: 24.5 mo	
Rwigema,2011	R	96	median:68.4	2 to 5	I:15–28; II:30–36		40.6% cetuximab	14	2-y LCR:57.8%(40-50 Gy);2-y LCR:31.7%(15-36 Gy)	Grade 3 acute toxicity:5.2%
					III: 40;IV: 44–50				2-y OS: 28.4%;median OS: 15 mo	
Vargo, 2012	R	150	median:68.4	5	40–50	/	47% cetuximab	/	/	Improved QoL
Lartigau, 2013	P	56		6	median:36	/	100% cetuximab	11.4	ORR:69.4%	Grade 3 toxicity:32.1%
									1-y OS: 47.5.0%;median OS: 11.8 mo, median PFS: 7.1 mo	
Vargo, 2014	R	28	median:70	5	40–44	/	50% cetuximab	14	1-y LRC: 51%	Grade ≥ 3 acute toxicity:0%
									1-y OS: 64%	
Bonomo,2014	R	17	median:66	5	median:30	/	11.8% chemotherapy	7.5	25% CR, 31% PR, 44% SD	Grade 3 toxicity: 6%
							5.88% cetuximab		Median PFS: 7 mo	
Kress,2014	R	85	median:68	5	median:30	29%	45% chemotherapy	17.3	2-y OS:24%	Grade 3 acute toxicity:2.4%
							25% cetuximab		2-y LRC: 28%	
Vargo,2015	P	50	median:70	5	40–44	/	100% cetuximab	18	1-y OS:40%, median OS: 10 mo	Grade 3 acute toxicity: 6%
									1-y LCR: 60%;1-y LRC:37%; median PFS: 7 mo	
Vargo,2017		414	≥ 40	5	40 (SBRT)	/	55% cetuximab or chemotherapy(SBRT)	28	2-y OS: 35.4% (IMRT) and 16.3% (SBRT) < 0.001	Grade ≥ 3 acute toxicity:12.2%(SBRT)
				33	60(IMRT)		84% cetuximab or chemotherapy (IMRT) Median OS: 13.3 mo (IMRT) and 7.8 mo (SBRT) < 0.001.			
Ansinelli,2018	R	45	NA	5	median:30	NA	NA	8.78	1-y OS: 37.76%; median OS: 9.23	Grade 3 acute toxicity:2.2%
McBride, 2021	P	62	NA	3	27	/	51.6% nivolumab	20.2	Nivolumab: ORR 34.5%; PFS:1.9 mo; 1-y PFS: 32.2%; median OS: 14.2 mo	SBRT: grade 3–5 toxicity 13.3%
									SBRT + nivolumab: ORR 29.0%; PFS: 2.6 mo;1-y PFS: 16.8%;median OS: 13.9 mo	
Pellizzon,2022	R	11	median:60	3	median:40	100%	NA	18	2-y OS: 80%; 4-y OS:53.5%	NA
									2-y DFS: 62.3%; 4-y OS:41.6%	
Huang, 2022	R	74	median:70	5	40–50		100% cetuximab	9	25.0% CR,41.7%PR Grade 3 acute toxicity:17.6%	
									2-y OS: 22.0%;median OS: 9 mo Grade ≥ 3 late toxicity:20.3%	

Abbreviations: OS, Overall Survival; LC, local control; LRC, locoregional control; DFS, disease free survival; PFS, progression free survival; CR, completed regression; PR, partial regression; SD, stable disease; ORR, objective regression rate

## Hyperfraction radiotherapy

Hyperfractionated radiotherapy (HFRT) is referred to two to three fractions are delivered each day, with a reduced dose per fraction equal to 1.1 to 1.2 Gy. The reduction of the dose per fraction may reduce the risk of late toxicity, despite an increased total dose [75].

RTOG 9610 is the first prospective muti-center clinical trial testing HFRT plus concurrent chemotherapy of 5-luorouracil (5-FU) and hydroxyurea. Among all the 79 analyzable patients, the estimated median survival is 8.5 months, with 1- and 2-year OS rates of 40.5% and 15.2%, respectively. The 5-year OS rate is only 3.8%. Overall, 38% and 18% of patients experienced grade 3 or 4acute toxicity, respectively. Six patients died during the therapy, resulting in 8% grade 5 toxicity. 19% grade 3 late toxicity was noted. Two grade 4 late toxicities (3%) occurred [76].

RTOG 9911 is another clinical trial utilizing the same radiation scheme, but the chemotherapy regimens were in substant of paclitaxel and cisplatin. 105 patients were enrolled and finally 99 patients are feasible for final evaluation. The median survival time was 12.1months, with a 1-year OS of 50.2% and a 2-year OS of 25.9%. Median PFS is 7.8 months with a 1-year PFS of 35% and a 2-year PFS of 15.8%., which was superior in RTOG9911 than RTOG 9610. Grade 3–5 acute toxicities were 49.5%, 23.2% and 5.1% respectively. And grade 3–5 late toxicities were 16.9%, 16.9% and 3.6%, respectively [77].

Since treatment-related toxicities, especially for acute toxicities were relatively high in concurrent chemotherapy with HFRT. Tao et al [78] reported that HFRT (60 Gy/50f, 1.2 Gy/f, bid) with weekly cetuximab was no significant different in OS, PFS or toxicities when compared with concurrent conventional chemotherapy. De-escalation was also evaluated. A group from Germany, showed that with doses of 30–36 Gy (1.5 Gy/f, bid), in combination with chemotherapy consisted of paclitaxel twice per week, the 1-year LRC, OS rate was 25% and 75%, respectively. Toxicity didn’t exceed grade 2 [79]. Rades et al. [80, 81] reported that using lower dose ranging from 36 to 44.4 Gy concurrent with paclitaxel, four of 6 patients survived over 12 months after re-irradiation. Only 1/6 patient developed into grade 3 toxicity. It seemed that lower dose resulted in lower incidence of toxicities, without compromising 1-year OS.

## Brachytherapy

Interstitial brachytherapy (iBT) is a form of internal radiation therapy where a sealed radiation source is placed inside or next to the tumor area. As rapid dose falloff beyond the implant part, surrounding normal tissues can be adequately spared from high doses of radiation. The rapid falloff in turn allows the delivery of a meaningful tumoricidal dose. Because brachytherapy treatment duration is typically much shorter than EBRT, it conquered the problem of accelerated repopulation of tumor colognes. So, in the past decades, brachytherapy has been investigated its role in rHNSCC [82].

### Low dose rate brachytherapy (LDR)

LDR has been the first and the most utilized technique in iBT as for its high LCR and relatively low treatment-related toxicity. Forty-five rHNSCC patients were treated with LDR alone (N = 22), surgery with LDR (N = 14), EBRT with LDR (N = 3) and triple treatment of all, EBRT and LDR (N = 6). The median dose of interstitial brachytherapy (iBT) was over 55 Gy. In the whole cohort, median PFS was 15 months, and LCR at 1 and 2 years were 50% and 37%, respectively. Median OS was 16 months, and OS at 2 and 5 years was 33% and 11%, respectively. Salvage surgery didn’t contribute to locoregional progression (P = 0.30). Severe retreatment morbidity was late toxicity which account for 1.2% in all the patients [83].

Two-hundred-twenty rHNSCC patients were treated by LDR of a median dose of 53 Gy, with or without salvage surgery, concurrent chemotherapy, or hyperthermia. Cumulative LCR at 2, 5, and 10 years were 69%, 51%, and 41%, respectively. The 2-, 5-, and 10-year DFS rates for the entire group were 60%, 33%, and 22%, respectively. The OS for the entire group at 2 and 5 years was 43% and 20%, respectively. However, further multivariate analysis indicated that prognostic factors, including tumor sites, age, gender, prior radiation dose, previous surgery, combination with chemotherapy and/or hyperthermia, and time interval between first radiation treatment and reirradiation, none of these were statistically significant in related with survival. 60% patients suffered from acute toxicities. Moderate to severe late complications occurred in 27% of the patients [82].

A retrospective review of the 51 rHNSCC patients at the M. D. Anderson Cancer Center was undertaken. All patients underwent neck dissection with complete resection and intraoperative placement of after loading brachytherapy catheters. The median iBT dose was 60 Gy delivered over an average duration of 5 days with an average dose-rate of 60 cGy/hour. The 2-year PFS rate was 58% and the median PFS was 59 months. The median OS was 101 months. The 2-year, 5-year, and 10-year OS rates were 69%, 56%, and 46%, respectively. Early adverse events were observed in 21 patients (39.6%) with 8 (15.1%) considered as grade 3 or 4 events. Late adverse events were observed in 19 patients (35.8%) with 6 (11.3%) considered as grade 3 or 4 events [84]. A prospective phase 1/2 trial involving 49 patients receiving surgery plus Cs-131 (surgery + Cs-131) treatment with a total dose of 60 Gy, grade 1 to 3 adverse events (AEs) occurred in 18 patients (37%), and grade 4 AEs occurred in 2 patients. Two-year PFS was 49% and 5-year OS was 31% [85].

### High dose rate brachytherapy (HDR)

HDR brachytherapy is another way allowing for optimization of treatment planning and more accurate dosimetry because of the ability leading to an advantage in both maximizing tumor control and minimizing complications. And on the other hand, HDR provide a shorter treatment duration [86].

Thirty patients of rHNSCC who received HDR with a median dose of 34 Gy (18–48 Gy) in twice daily fractions of 3 to 4 Gy per fraction. LCR was achieved in 69% of implanted sites.

DSS at 1 and 2 years was 54% and 45%, respectively. OS at 1 and 2 years was 56% and 37%, respectively. Grade 3/4 late complications occurred in 16% of the patients. No fatal complications occurred [87]. Wiegand et al. showed that HDR of 20-33 Gy with a fraction dose of 2-3 Gy in inoperative rHNSCC patients. The 1-year OS and 2-year OS were 41% and 18%, respectively [88]. Another retrospective study presented that HDR of curative intent or palliative intent. One-year rates of LC, RC, DMFS, and OS were 55%, 62%, 94%, and 77%, respectively. Patients treated with curative intent had 2-year rates of LC, RC, DMFS, and OS of 73%, 55%,100%, and 56%, respectively. When stratifying for curative versus palliative intent, LC (P = 0.011) and OS (P = 0.0084) were both significantly improved with curative intent. 33% had grade 3 to 4 late toxicities. Curative-intent HDR brachytherapy reirradiation can provide excellent local control and encouraging OS [89].

HDR has also been used in combination with salvage surgery. The group from Memorial Sloan-Kettering Cancer Center analyzed 30 rHNSCC patients treated by HDR. Eighteen patients underwent surgical resection followed by HDR, 3 patients were treated with combined EBRT and HDR, and the remaining 9 were treated with HDR alone. The dose and fractionation schedules used were 3.4 Gy twice per day (b.i.d.) to 34 Gy for postoperative cases, 4 Gy b.i.d. to 20 Gy when combined with 40-50 Gy external beam, and 4 Gy b.i.d. to 40 Gy for definitive treatment. The 2-year LC and OS for the entire group were 71% and 63%, respectively. Patients treated with surgical resection and HDR had an improved 2-year LC compared to the patients treated with HDR+/-external beam radiation alone (88% vs. 40%, p = 0.05). Grade 2 and grade 3 complications were noted in five patients, all observed in the postoperative HDR group [90]. A European group also evaluated the role of HDR in addition to resection with a median single dose of 3 Gy applied in 2 daily fractions (interfraction interval ≥ 8 h), and a median total physical dose of 30 Gy. Outcomes were similar: 2-year OS of 13 recurrent patients was 65.3% and the mean OS was 22.8 months [91].

A prospective study compared the efficacy between 3DRT and HDR in rHNSCC patients. Total of 64 patients with head and neck cancer recurrence were randomly assigned at a 1:1 ratio to receive either 3DRT (50 Gy/25 fractions) in the control group or HDR (30 Gy/12 fraction). The OS rate of patients treated with HDR at 1 and 2-years was74% and 67%, respectively, compared to 3DRT group − 51% and 32%, respectively (P = 0.002). LCR at 1- and 2-years in patients who received HDR was 77% and 63% compare with 47% and 25%, respectively, for the patients who received the 3DRT (P < 0.001). Grade 3–4 acute toxicity occurred in 34.4% in HDR group and 54.8% in 3DRT (P = 0.102). In the 3DRT group, severe late toxicity was determined in 11 patients (35.5%), and in the HDR group, in 1 patient (3.1%) (P = 0.001). There was no grade 5 toxicity in both groups [92].

### Pulse dose rate brachytherapy (PDR)

The main theoretical advantage of PDR-brachytherapy is that it combines the biological advantages of LDR-brachytherapy with the technological advantages of the HDR after loading method.

Strnad et al. showed that 34 of 43 rHNC patients received interstitial PDR brachytherapy (D_REF_=20–60 Gy) as part of their curative treatment regimen alone or in combination with external radiation. Meanwhile 9 patients were implanted for palliative purposes. The pulses were delivered 24 h/day with a time interval of 1 h between two pulses. The dose per pulse (dp) ranged from 0.4 to 0.7 Gy. 37% patients also received cisplatin or carboplatin with 5-Fu during the time of the PDR. 30% patients received EBRT in a dose range from 20 to 67 Gy. LCR rate was 79% and DMFS rate was 12%. The 2-year LRFS rates, DFS rates, and OS rates for all patients were 68%, 62%, and 49%, respectively, and for patients treated with curative intention they were 80%, 77% and 66%, respectively. Soft tissue necrosis was the only serious side effect seen in 4.7% patients [93]. In 2014, the same group presented 51 patients with rHNC by using the same scheme. LCR at 2 and 5 years were 71% and 57%, respectively. Comparing results of salvage PDR brachytherapy with or without simultaneous chemotherapy, the 5-year LRFS rates were 78.9% vs. 38.5% (p = 0.01), respectively. A total of 9/51 (17.7%) and 6/51 (11.8%) patients developed soft-tissue necrosis or bone necrosis, respectively, but only 2% of patients required surgical treatment [94].

Kremplien et al. reported a retreatment of 14 locally rHNSCC by combined brachy-chemotherapy using frameless image-guided 3D interstitial brachytherapy. The 1- and 2-year LCR were 78% and 57%, respectively. The actuarial 1- and 2-year OS rates were 83% and 64%, respectively. The median survival was 28 months [95].

## Proton and heavy ions

Recently, the use of proton or heavy ion therapy has been shown to be beneficial by harnessing the power of the Bragg peak, where most of the dose is deposited in the target followed by a steep dose decrease, thereby limiting dose to normal tissues outside the treatment field. So compared to conventional photo radiotherapy will improve the capability of protecting surrounding tissue.

### Proton beam radiotherapy (PBRT)

A retrospective analysis of 92 consecutive patients were treated by curative intent PBRT, 52.2% patients received systemic chemotherapy. The median PBRT dose was 60.6 Gy (relative biological effectiveness, [RBE]). The cumulative incidence of locoregional failure at 12 months, was 25.1%. The actuarial 1-year DMFS and OS rates were 84.0% and 65.2%, respectively. Acute toxicities of grade 3 or greater included mucositis (9.9%), dysphagia (9.1%), esophagitis (9.1%), and dermatitis (3.3%). Grade 3 or greater late skin and dysphagia toxicities were noted in 6 patients (8.7%) and 4 patients (7.1%), respectively. Two patients had grade 5 toxicity due to treatment-related bleeding [96].

Another report that in 61 rHNC patients treated by reirradiation with PBRT, 47.5% received salvage surgery prior to PBRT, in whom 70.5% were with gross disease. The median dose was 66 Gy (RBE) and for gross disease was 70.2 Gy (RBE). 27.9% patients received concurrent chemotherapy. The 2-year OS was 32.7%, and the median OS was 16.5 months. The 2-year cumulative incidence of local failure with death was 19.7%. Grade ≥ 3 toxicities were seen in 38.3% patients, including 14.7% acutely and 24.6% in the late setting. More importantly, KPS ≤ 70, the presence of a gastrostomy tube before reirradiation, and an increasing number of previous courses of radiation therapy were associated with a greater hazard ratio for death. A cutaneous primary tumor, gross residual disease, increasing gross tumor volume, and a lower radiation dose were associated with a greater hazard ratio for local failure [97].

Beddok et al. compared the PBRT with IMRT in 55 rHNSCC patients. Patients received IMRT (52.2%) or PBRT (47.8%) at a median maximum dose to the CTV of 66 Gy. For the IMRT group, 1- and 2-year local failure free survival (LFFS) was 12.1% and 12.1%, respectively. For the PBRT group, 1- and 2-year LFFS was 50.0% and 22.2%, respectively. For the IMRT group, 1- and 2-year OS was 27.3% and 27.3%, respectively. For the PBRT group, 1- and 2-year OS was 91.7% and 55.6%, respectively.

For the whole cohort, 1- and 2-year DFS was 30.4%, DFS was significantly longer in the PBRT group (p = 0.031). Five patients (21.7%) experienced grade 3 acute toxicity (dysphagia, dermatitis, and mucositis). The comparison between patients in the IMRT and PBRT groups revealed that severe dysphagia (grade > 2) was significantly more frequent in the IMRT group (p = 0.006). Severe dermatitis was also more frequent in the IMRT group than in the PBRT group, although the difference was not statistically significant (p = 0.093). In contrast, hearing loss was significantly more frequent in the PBRT group than in IMRT group (p = 0.004), This result could be explained by the slightly different locations of the tumors treated with PBRT (skull base) and those treated with IMRT. Main late grade ≥ 2 toxicities were dysphagia and trismus [98].

An expanded cohort of 242 rHNSCC patients received re-PBRT treated either by fractionated PBRT (median dose of 70 cobalt gray equivalents, CGE) or by quad shot PBRT (median dose of 44.4 CGE). The 1-/ 2-year LCR was 71.8% and 63%, respectively in fractionated group in comparison with 61.6% and 52.5%, respectively in quad shot group. There was a total of 73 grades 3 and 6 grade 4 early toxic effects. There were 79 potential grade 3, 4 grade 4, and 5 grade 5 late toxic effects [99].

A study from Japan reported a group of recurrent oral cancer patients treated by PBRT with median re-irradiation dose of 50 GyE in 24 fractions along with weekly concurrent intra-artery cisplatin infusion. In total 34 patients, 22 patients (65%) achieved a CR and 12 patients (35%) PR at the primary tumor. Clinical outcomes have been better with 1-year/2-year OS rates were 62% and 42%, and the 1-year/2-year LC rates were 77% and 60%, respectively. Grade ≥ 3 toxicities were leukopenia in six patients (18%); thrombocytopenia in nine patients (24%); oral mucositis in 11 patients (32%); radiation dermatitis in 10 patients (29%) and dysphagia in 12 patients (35%). 3% patients had late toxicity of osteonecrosis. This study showed that lower dose of 50 GyE with weekly concurrent cisplatin intra-artery infusion would be feasible without compromising survival benefit or toxicities [100].

### Heavy ion beam radiotherapy

A group from Heidelberg investigate the efficacy and safety of second carbon ion radiotherapy(CIRT) in rHNC patients. This study involved 229 rHNC patients. The median radiation therapy interval was 3.9 years, and patients received a median dose of 51 Gy (RBE) in 3 Gy (RBE) fractions. The median LPFS and OS were 24.2 months and 26.1 months, respectively. Serious acute toxicity (grade ≥ 3) was 3.0%. Late toxicities over grades 3 was 14.5% [101].

A Japanese group retrospectively reviewed 56 rHNC patients who previously treated by conventional RT, underwent re-CIRT. The most used protocol was 57.6 Gy (RBE) in 16 fractions (n = 23, 41.1%). Surgery preceded re-RT in three patients (5.4%). One patient with malignant melanoma received concurrent chemotherapy. The 2-year LC, PFS, and OS rates were 66.5%, 36.9%, and 67.9%, respectively. The median follow-up time was 28 months. Two patients (3.6%) developed grade ≥ 3 acute toxicities, and 14 (25.0%) developed grade ≥ 3 late toxicities. A single patient had confirmed grade 5 dermatitis with infection [102].

Hayashi et al, reviewed 48 rHNC patients who received re-CIRT. The median dose of initial CIRT and that at re-irradiation were 57.6 Gy and 54.0 Gy (RBE), respectively. Concurrent chemotherapy was not applied in this study. Five patients (10.4%) developed Grade 3 acute toxicities and 18 (37.5%) developed Grade ≥ 3 late toxicities, including Grade 5 central nervous system necrosis in one patient. The 2-year LC, LRC, PFS, and OS rates were 40.5%, 33.5%, 29.4%, and 59.6%, respectively [103].

## Organ at risk (OAR) in re-irradiation in HNSCC

Though re-irradiation is an altherantive for rHNC patients, the late toxicity to surrounding OAR is a major limitation on dose escalation. Limited studies have published on cumulative doses to normal tissues and dose constraints in the re-irradiation settings, including spinal cord, brainsterm, carotid arteries, temporal lobe, and bone structures, i.e. mandible [104].For spinal cord, Nieder et al [105] reporte the cumulative biological effective dose (BED) ≦ 120Gy_2_, and the treatment interval is no shorter than 6 months and the dose of each course is ≦ 98Gy_2_, there was no case of radiation myelophathy(RM).And even BED escalated to 135 Gy, the risk of RM appears small. On the other hand, Ahlawat showed that the PRV spine and PRV brainterm cumulative doses were 50 and 54 Gy,respectively [106]. In SBRT study, the maximum dose to spinal cord, branstem, and optic nerve were 21 Gy, 37 Gy, and 34 Gy, respectively [67]. Carotid blowout is another servere late toxicity in rHNC re-irradiation, as for it’s the main cause to fatal hemorrhage. It was reported that the lifetime dose of radiation to carotid blowout is 126 Gy [107–109]. Temporal lobe necrosis (TLN) is a common late complication resulting in cognitive disorder, so the dose to TL is registered a cumulative BED < 150Gy_2.5_ [110]. When considering the osteoradionecrosis (ORN), Bots et al. suggested that the median dose of 114 Gy (rang, 94-30 Gy) might lead to 5.8% ORN following the re-irradiation. And the dose to the mandible for developing mandible ORN was 104-128 Gy [108]. A group from US presented a systematic approach to the reirradiation process regarding of tissue repair when limite the dose to OARs. They used a model combining the cumulative BED and discount factors to predicit dose constrains of OARs, including: spinal cord, brainterm, retina and esophagus [111].

## Conclusion

Recurrent head and neck or second primary tumor are a major failure pattern of head and neck malignancies which account for 40% in all the HNC patients. Salvage surgery was a definitive treatment. However, salvage surgery is not feasible for all the rHNC patients. With the development of radiotherapy technology, the effect on rHNC patients has been extensively evaluated and reirradiation is associated with better prognosis. And even post-operative radiotherapy also results in long-term survival. Different radiotherapy techniques such as, IMRT, VMAT, SBRT, hyperfraction radiotherapy, SBRT, brachytherapy or proton/heavy ion therapy showed their own advantages and should be carefully utilized in certain circumstances. And the treatment-related toxicity is an important reason for treatment-related mortality which compromised survival benefit. Concurrent chemotherapy showed no significant in extending OS but resulted in better local control, cetuximab is an alternative for those who cannot undergo chemotherapy. Immunotherapy in combination with re-irradiation should be further evaluated. However, this review is mostly based on retrospective studies, lacking of large prospective clinical trials has certain limits on the supporting evidence strength and validity. And more prospective trials are needed to compare those different modalities for better understanding of its efficacy and safety.

## Data Availability

Not applicable.

## Abbreviations

AEs: adverse events
CGE: cobalt gray equivalents
CIRT: carbon ion radiotherapy
DFS: disease free survival
DMFS: distant metastasis free surviva
DCR: disease control rate
DSS: disease-specific survival
DRRT: definitive reirradiation
ECE: extracapsular extension
FFM: freedom from metastases
HFRT: hyperfractionated radiotherapy
HDR: high dose rate brachytherapy
HNSCC: head and neck squamous carcinoma
rHNC: recurrent head and neck cancer
IGRT: image-guided radiotherapy
IMRT: intensity modulated radiotherapy
ICIs: immune checkpoint inhibitors
iBT: interstitial brachytherapy
LRC: locoregional control LCR local control rate
LRRFS: locoregional recurrent free survival
LRFS: local recurrent free survival
LFFS: local failure free survival
LDR: low dose rate brachytherapy
ORR: objective response rate
OARs: organs at risks
OS: overall survival
PFS: progression free survival
PRRT: postoperative reirradiation
PDR: pulse dose rate
PBRT: Proton beam radiotherapy
RR: response rate
RB: relative biological effectiveness
SBRT: stereotactic body radiotherapy
VMAT: volumetric modulated arc therapy
5-FU: 5-fluorouracil
BED: biological effective dose
TLN: temporal lobe necrosis
ORN: Osteoradionecrosis
RM: radiation myelophathy
